# Is it Possible to Assess the Two-Domain Definition of the Broad Autism Phenotype Using the Available Measurement Tools?

**DOI:** 10.1007/s10803-021-05158-7

**Published:** 2021-06-29

**Authors:** M. Godoy-Giménez, A. González-Rodríguez, F. Cañadas, A. F. Estévez, P. Sayans-Jiménez

**Affiliations:** 1 Department of Psychology, University of Almeria, 04120 Almería, Spain; 2 CERNEP Research Centre, University of Almeria, 04120 Almería, Spain

**Keywords:** Broad autism phenotype, Test content, Expert judgment, BAPQ, SRS, AQ

## Abstract

Although, the operationalization of the autism spectrum disorder has been updated around two domains, the broad autism phenotype (BAP) one has not. Additionally, the items of the three common BAP measures, the Broad Autism Phenotype Questionnaire (BAPQ), the Autism Quotient, and the Social Responsiveness Scale (SRS), remain organized around a non-consensual number of factors. We explored whether the items of these measures matched with the two-domain operationalization through a parallel analysis, which has suggested two main components, and two expert judgments which have assessed item wording, relevance, and construct representativeness. A remaining pool of 48 BAP-relevant items suggested a possible under-representation of two subdomains. Despite the relevance of all the BAPQ items, only the SRS ones tapped in all subdomains.

**Supplementary Information:**

The online version contains supplementary material available at 10.1007/s10803-021-05158-7.

## Introduction

The broad autism phenotype (BAP) has been described as a set of subthreshold features qualitatively similar to those existing in autism spectrum disorder (ASD), which is continuously distributed and spreads beyond ASD family members into the general population (Constantino & Todd, 2003; Hoekstra et al., 2007; Hurst et al., 2007; Stewart & Austin, 2009). As pointed out by numerous authors (e.g., Morrison et al., 2018; Wainer et al., 2011), BAP traits correspond directly to the primary characteristics of ASD: Social Communication and Social Relation Impairment (SCI) alongside a Pattern of Restricted Repetitive Behaviours and Interests (RRBs; American Psychiatric Association [APA], 2013). Even though the definition of ASD has been updated over time, the most popular BAP definition has barely changed and has not been revised according to the most recently updated definition of ASD (APA, 2013). This has generated a discrepancy between ASD and BAP conceptualizations that, in our opinion, needs to be addressed.

The reharmonization of the BAP and ASD constructs will highly contribute to the field as it will operationalize the BAP in the spectrum, increasing its understanding and, providing further information regarding how core autistic deficits are expressed differentially on each severity level. Likewise, it will also help to uncover autistic genetic mechanisms (Gaugler et al., 2014; Robinson et al., 2011) by exploring the presence of BAP in parents of children diagnosed with ASD. In the same way, it will also increase the understanding of the developmental path of autism in elder adults (Stewart et al., 2018) which has not been very studied yet. Finally, we can as well take advantage of BAP samples to test assessment and intervention procedures before applying them to clinical and more sensible samples.

On the other hand, accepting that both constructs essentially represent the same spectrum of traits to a different degree, could bring many advantages for several populations. As such, uncertain cases of those individuals who do not have a clear ASD diagnosis but have shown highly impaired BAP behaviors and, in lower intensity, independent and functional adults with some core autistic behaviors that impoverish their social interactions, as well as those capable of camouflaging or smoothing specific deficits (developing ways to cope with them; Hull et al., 2017; Livingston et al., 2019; Mandy, 2019), could also benefit from well-established autism assessment protocols and interventions which will represent, indeed, a clinical milestone.

In this regard, the reconciliation of BAP and ASD will drive certainly to restore any previous measurement process. According to the Standards (2014), whether two tests have defined the same construct differently and, thus, contain different internal structures, those tests are assessing essentially two different things. The BAP, as the autistic phenotype, has been proposed to share ASD internal structure, and, consequently, tests that aim to measure the BAP should be developed upon an updated two-factor internal structure. Conducting studies to establish conclusions on ASD populations through BAP samples necessarily imply the existence of a measurement tool capable of measuring an updated BAP operationalization. For all the abovementioned reasons, in this work, we aimed to confirm whether it is possible to integrate the items of the most frequently used tests for assessing the BAP to provide a BAP measure that would be aligned with the current operationalization of ASD.

### The Outdated Operational Definition of the BAP

The BAP was first reported by Leo Kanner (1943) who observed that the parents of children diagnosed with ASD presented subtle expressions of autistic-like traits such as an obsession with details, social awkwardness, and rigid behaviours. Later, more formal investigations conducted by Piven et al., (1997a, 1997b) identified autistic-like core deficits in first-degree relatives of people diagnosed with ASD. Those deficits established the foundations for the first operational definition of BAP, which was developed by Hurley et al. (2007), who, after reviewing outcomes of the studies conducted in the previous two decades, defined the BAP as a set of subclinical personality characteristics and language deficits clustered around three main domains; aloof personality, rigid personality, and pragmatic language impairment paralleling its definition with that proposed for ASD by the DSM-IV-TR (American Psychiatric Association [APA], 2000).

According to the literature, an aloof personality involved a lack of social responsiveness (Constantino et al., 2006), reduced social abilities (Wheelwright et al., 2010), and scarce social engagement (Whitehouse et al., 2010). Likewise, a rigid personality was manifest in behavioural rigidity, a tendency toward perfectionism, stubbornness, and stereotyped behaviours (e.g., Losh et al., 2008; Murphy et al., 2000; Narayan et al., 1990). Finally, pragmatic language deficits were related to supra-linguistic aspects such as problems in respecting turn-taking in speaking, becoming side-tracked in conversations, and difficulties in maintaining the topic of conversations (Seidman et al., 2012).

Although this is now a more mature field, there is still no universal agreed operationalization of the BAP, but rather a quantitative and qualitative amalgam of traits that vary according to the measurement method used to assess the phenotype (Wainer et al., 2011). Those varied features have been, sometimes, difficult to cluster so as to conform to a consensual structure for the BAP enabling to reinsert it inside the autism continuum. In light of more recent research, it makes no sense that the phenotypic expression of ASD, which had emerged for improving the knowledge about its aetiology, would diverge from its mother disorder and, thus, consensus could be reached in the conceptualization of the BAP by aligning it with the updated definition of the ASD (APA, 2013). According to this definition, ASD has been defined as a continuum of increasing severity. Some authors have previously argued in favour of this idea, associating the lesser, non-clinical expressions of autism, with the BAP (Bolton et al., 1994; Constantino & Todd, 2003; Piven & Palmer, 1999). In this regard, some studies have already proposed that the BAP should comprise only two characteristic traits, these being conceptualized as both social and non-social expressions of the BAP, where social traits have included both social impairment in social relations and in social communication; whilst non-social traits have constituted a rigid personality (Morrison et al., 2018; Sasson et al., 2013b).

### The Measurement of the BAP

Although the BAP has traditionally been assessed through structured and extensive interviews designed to evaluate personality (i.e., M-PAS-R; Piven et al., 1994), the use of brief psychometric self or informant-reports has increased, reducing time costs and enhancing objectivity. Among these questionnaires, the Autism Spectrum Quotient (AQ; Baron-Cohen et al., 2001), the Social Responsiveness Scale (SRS; Constantino & Gruber, 2005), and the Broad Autism Phenotype Questionnaire (BAPQ; Hurley et al., 2007) have been the most widely used (for a review see, Ingersoll & Wainer, 2014).

Despite having plenty of benefits, researchers and clinicians should be particularly careful regarding the theoretical and empirical evidence supporting the interpretation of these test scores. In particular, following the changes in the operationalization of ASD from three to two general domains (APA, 2013), it might be advisable to draw special attention to different sources of validity evidence such as the internal structure or test content. Adverse evidence would imply the need to question the interpretation of the test scores (American Educational Research Association, American Psychological Association, & National Council on Measurement in Education [AERA, APA, & NCME], 2014). As stated above, there has been a lack of correspondence between the operationalization of the BAP (understood as the subclinical expression of autism which includes both impairment in social interactions and rigid behaviours) and the content of the tests most frequently used for its evaluation. Of equal importance is the fact that this lack of correspondence could also be observed between the operationalization of the construct and the internal structure of these tests.

The three aforementioned measures have reflected this lack of correspondence. Thus, the AQ—which was originally designed for assessing autism in adults with typical-range IQs—comprises five content-domains and five factors: Social Skills, Attention Switching, Attention to Detail, Communication, and Imagination (Baron-Cohen et al., 2001). In spite of contradictory data in the literature, some studies have supported the clustering of the five subscales into a three-factor model: Social skills, Details/patterns, and Communication/mindreading (English et al., 2019; Hurst et al., 2007; Russell‐Smith et al., 2011). Similarly, the second adult version of the SRS (SRS-2) measures autism in adults with typical development and includes five subscales: Social Awareness, Social Cognition, Social Communication, Social Motivation, and Restrictive and Repetitive Behaviors (Bruni, 2014; Constantino & Gruber, 2012). It includes two subscales compatible with the DSM-5: Social Communication and Interaction, and Restricted Interests and Repetitive Behaviours. Scores on these subscales have facilitated the comparison of symptoms with DSM-5 diagnostic criteria for ASD. These comparisons could help to determine whether a person meets the most current diagnostic criteria for ASD (Constantino & Gruber, 2012). As support for this idea, Frazier et al. (2014) observed that SRS-2 subscales could be encompassed by the following two-structured factors: Social Communication/Interaction (SCI), and Restricted/Repetitive Behavior (RRB; Frazier et al., 2014). Finally, the BAPQ, the tool originally designed for assessing the BAP in parents of children diagnosed with ASD, includes three factors: Aloofness, Pragmatic Language Deficits, and Rigidity (for a review, see Hurley et al., 2007; Sasson et al., 2013a).

Despite the diverse specifications of these tests, some authors have suggested that these all could serve to assess the same underlying BAP structure. In an attempt to search for the similarities between these tests and to obtain an empirically-based latent structure of the BAP in a non-clinical sample, Wainer et al. (2011) conducted a conjoint exploratory factor analysis by collapsing the AQ, the SRS-A (adult self-report version; Constantino & Gruber, 2005), and the BAPQ subscales. They concluded that the subscales could conform to a three-factor structure similar to that proposed for ASD by the DSM-IV-TR (APA, 2000). These three factors, named Aloof Personality, Pragmatic Language Difficulties, and Rigid Personality, matched with the BAPQ factors and with one of the most accepted descriptions and operationalizations of the BAP (Piven et al., 1997a, 1997b).

In view of their findings, Wainer et al. (2011) also discussed their resulting BAP structure and suggested that more research was needed to clarify whether the core areas of impairment observed in ASD (and, by extension, the BAP as its non-clinical expression) could be grouped into two core domains. This was in line with recent literature that has suggested the need for a new conceptualization of the phenotype aligned with the current definition of ASD (APA, 2013), centred around two core domains: social and non-social areas (Morrison et al., 2018; Sasson et al., 2013b).

To take the work of Wainer et al. (2011) one step further, the present study aimed to extend and revise their findings by exploring the connection between the AQ, the BAPQ, and the SRS-2 subscales with an updated conceptualization of BAP according to two main dimensions or domains: Deficits in Social Communication and Social Interaction (hereafter, SCI BAP) and Restricted, Repetitive Patterns of Behaviours, Interests, or Activities (hereafter, RRB BAP). To this end, our study firstly explored the internal structure resulting from applying parallel analysis (Horn, 1965) to the set of total scores of each subscale of the mentioned tests (first objective, parallel analysis). Secondly, we took the first step towards bridging the gap between the operationalization and measurement of the BAP by selecting the most relevant items for its measurement. For this second objective, a group of collaborators from our research lab allocated the items of these questionnaires, according to their content, to the seven ASD subdomains (Loevinger, 1957). Furthermore, they verified their itemmetric properties (see Angleitner et al., 1986; Grant & Davis, 1997).

Finally, we recollected quantitative information regarding content validity (Lynn, 1986). For achieving our third objective, a broad group of experts evaluated the relevance (Ebel & Frisbie, 1972; Haynes et al., 1995) and the representativeness (Haynes et al., 1995; Lynn, 1986) of the selected items for assessing BAP features based on the updated operationalization aligned with the current definition of ASD (Armstrong et al., 2005; Beck & Gable, 2001; Delgado-Rico et al., 2012).

Following the results presented in Wainer et al. (2011), we hypothesized, that the subscales of the AQ, the BAPQ, and the SRS-2 could be clustered into three components of variance that could correspond to the original conceptualization of BAP (first objective; Hurley et al., 2007; Piven, et al., 1997a, 1997b). Further, we expected to find some problems regarding the formal aspects of items (second objective; see Angleitner et al., 1986). For instance, some items could present high levels of social desirability, low levels of self-reference, lack of concreteness or be difficult to understand. In addition, since items stem from different tests with different operationalizations of the BAP (in the case of BAPQ) and ASD (in the case of AQ and SRS-2), we hypothesized that their relevance for assessing one of the seven BAP subdomains could equally affect their final selection (third objective). For example, Item 11 SRS-2 states “I have got self-confidence” which could not be relevant since it did not capture any of the key contents of the seven BAP subdomains. Finally, since the items were constructed within the framework of previous BAP or ASD definitions, we anticipated that some of the seven BAP subdomains could be under-represented (third objective).

## Method

### Participants

#### Parallel Analysis

A total of 349 undergraduates [26.1% men, M (SD) = 21.56 (4.63); 73.9% women, M (SD) = 20.88 (4.01)] completed a booklet which contained the questionnaires. Participants were recruited from the Degree of Psychology of the University of Almeria through incidental and snowball sampling. Specific data on socioeconomic status and ethnicity were not recorded. None of the students reported severe or genetic conditions or a history of psychiatric disorders.

#### Item Selection and Assignment According to Their Content and Itemmetric Properties

Five collaborators of our research lab took part in this phase of the study (FC, AFE, MGG, and AGR were experts in neuropsychology and PSJ in psychometrics). They were purposely selected due to their expertise in the core fields associated with our intended objectives (i.e., autism-related traits and test construction).

#### Expert Judgment

Twenty judges (18 professionals and two academic experts) participated in this second phase. The criteria for selecting them were, in the case of professionals, to have been working with ASD people and their families during the past five years. Academic experts must have completed a doctoral dissertation and have more than five published articles on the subject. Moreover, to ensure the representativeness of the sample of experts (Davis, 1992), we developed a grid with fields related to ASD, in both professional and academic areas (see Table 1). Following the authors’ recommendations (Gable & Wolf, 1993; Tilden et al., 1990) and due to the large pool of items, an initial sample of 32 experts (24 professional experts and eight academic experts) were selected. After making contact with them, 23 agreed to participate in the study. Three of them were subsequently deleted from the data due to missing information in their booklets. Regarding formal education of final grid of experts, eleven of them had studied a Degree in Psychology, two have a Degree in Speech Therapy, four had a Degree in Special Education, one did a Degree in Pedagogy, and two had higher studies in Neuropaediatry.

**Table 1:** Grid of ASD related areas of expertise covered in Expert Judgment

Professional experts (n = 18)					
Health field (n = 11)		Social field (n = 2)		Educational field (n = 5)	
Early care		Professional workers in autism associations	n = 2	School counsellors	n = 2
Speech therapist	n = 2				
Child neuropsychologist	n = 2				
Child psychologist	n = 2				
Neuropaediatric Unit	n = 1			Workers in therapeutic pedagogy	n = 3
Child psychologist (psychologist specialist in clinical psychology)	n = 4				

Academic experts (n = 2)
Expert in educational and developmental psychology (n = 1)
Expert in clinical child psychology (n = 1)

### Instruments

#### Parallel Analysis

##### The Broad Autism Phenotype Questionnaire (Spanish Self-Report Version)

The BAPQ-SP (Godoy-Giménez et al., 2018; Hurley et al., 2007) was a 36-item screening questionnaire specifically designed to assess the BAP in relatives of people diagnosed with ASD in the Spanish population. BAPQ items were grouped into three subscales, which corresponded with the original BAP core factors (Hurley et al., 2007; Piven et al., 1997a, 1997b): aloofness, rigidity, and pragmatic language problems. The correspondence of the items and the subscales as well as the reversed items can be found in Hurley et al. (2007).

##### The Autism Quotient

The AQ (Baron-Cohen et al., 2001) was a 50-item self-report scale designed to identify adults diagnosed with high-functioning autism and standard intelligence. AQ items were grouped into five subscales: Social Skill, Attention Switching and Attention to detail, Communication, and Imagination. However, the test only provides a total score; the correspondence of the items and the subscales as well as the reversed items can be found in Baron-Cohen et al. (2001). The items of the AQ were adapted to the Spanish language following the guidelines proposed by Muñiz et al. (2013) and the International Test Commission Guidelines on Adapting a Test (http://www.intestcom.org) with the assistance of an official translator.

##### The Social Responsiveness Scale-2 (Adult Self-Report Version)

The SRS-2 (Constantino & Gruber, 2012) was a 65-item ordinally quantitative test for examining the severity of autistic traits in adults. SRS-2 items were grouped into five factors: Social Awareness, Social Cognition, Social Communication, Social Motivation, and Restricted Interests and Repetitive Behaviours; the correspondence of the items and the subscales, as well as the reversed items, can be found in Constantino and Gruber (2012). It also included two subscales compatible with the DSM-5: Social Communication and Interaction, and Restricted Interests and Repetitive Behaviours. As described previously, the items of the SRS-2 were adapted to the Spanish language following the guidelines proposed by Muñiz et al. (2013) and the International Test Commission Guidelines on Adapting a Test (http://www.intestcom.org) with the assistance of an official translator. At the time of conducting this study, there was no Spanish adaptation of the test available; the official Spanish SRS-2 adult self-form was published one year later (Constantino, 2017).

#### Item Selection and Assignment According to Their Content and Itemmetric Properties

##### Assignment of Items to ASD/BAP Subdomains

Experts received two text documents by email, the first of which listed the seven subdomains of the updated BAP operationalization and their definitions (see supplementary material Table 1), and a second that included the items of the three questionnaires in this order: BAPQ, SRS-2, and AQ.

##### Itemmetric Analysis

Later, the experts also received an excel document containing the results from their previous assignment of the items. On this occasion, the selected items of the three questionnaires were randomized and included as a common pool of 121 items (39 items from the AQ, 36 items from the BAPQ, and 46 items from the SRS-2) without any reference to the original factor or questionnaire to which they belonged. The experts had to rate from 1 to 4 (for example, 1 = *not clear,* 2 = *somewhat clear,* 3 = *quite clear,* and 4 = *very clear*) each item according to the following properties: clarity (item was accurate and excluded double negations, adverbs incongruent with the rating scale, and multidimensionality), comprehensibility (readers could understand the item at the outset), concreteness (each item referred to only one idea), degree of self-reference (responses to the item could be expressed based on a person’s perception of him/herself), and evaluation of the items (responses to the item could be influenced by social desirability).

#### Expert Judgment

The documentation for Expert Judgment included (i) a cover letter with information on the research group and the scope of the study; (ii) the updated BAP operationalization around two core domains and seven subdomains aligned with the updated ASD definition in the DSM-5 (APA, 2013; see supplementary material Table 1); (iii) a brief explanation and examples of BAP domains and subdomains. It also contained the instructions and variables targeted in this study (all files included in Expert judgment 2 can be consulted in supplementary material Document 1 and Document 2). Following authors’ recommendations (Almanasreh et al., 2019; Lynn, 1986) quantitative findings of content validity were collected for assessing the relevance of each item for the subdomain and representativeness of the BAP construct. Both of these aspects were rated by the experts using a Likert-type ordinal scale with four possible responses (for relevance: 1 = not relevant, 2 = somewhat relevant, 3 = quite relevant, and 4 = very relevant; and for representativeness: 1 = very poorly represented, 2 = poorly represented, 3 = well represented, and 4 = very well represented). Finally, we also took into account other variables beyond the scope of this study (experts were asked about the adequacy of the items to the objectives of a new test and whether the items were susceptible to differential functioning between targeted populations).

### Procedure

#### Parallel Analysis

Two booklets containing the items from the AQ, the BAPQ, and the SRS-2, together with two other questionnaires beyond the scope of this study,^1^ were administered to the sample of students. Although the participants were given as much time as they needed to complete the questionnaires, the testing phase lasted approximately 50 min (for further information, see Godoy-Giménez et al., 2018).

#### Item Selection and Assignment According to Their Content and Itemmetric Properties

First, a group meeting was held where the collaborators were given prior instructions about the entire procedure, the theoretical foundations of each questionnaire, and the updated BAP operationalization. Then, each member independently allocated the items to the seven subdomains of that updated BAP operationalization.^2^ They were also asked to make a note of items that did not fit into any subdomain. Later, the five collaborators shared their ideas and discussed any discrepancies about item assignations. Finally, they independently assessed the itemmetric properties of all the items of the three questionnaires.

#### Expert Judgment

The documentation was sent to the experts by ordinary mail. They were informed that they had two weeks to complete the whole task and return it to us by a pre-addressed postage-paid envelope. The experts were assured that they could rely on our assistance during the assessment process, although none of them required it.

### Data Analysis

#### Parallel Analysis

We conducted a parallel analysis of principal components (Horn 1965) on the total raw scores of each of the AQ, BAPQ, and SRS-2 subscales. The analyses were conducted using Psych package (Psych 1. 9.12.31, 2020) in R software version 3.6.3 (R Core Team, 2020).

#### Item Selection and Assignment According to Their Content and Itemmetric Properties

Following previously established guidelines (Angleitner et al., 1986; Osterlind, 1998) the items were selected according to their itemmetric properties; thus, only items that showed expert agreement (that is, with averages ≥ 3.8) on all the variables assessed were incorporated into the test. Since these questionnaires have been used to discriminate among people with different levels of BAP severity (e.g., Baron-Cohen et al., 2001; Constantino & Todd, 2003; Hoekstra et al., 2007; Sasson, et al., 2013b; Shi et al., 2015), or for predicting BAP-related variables (e.g., Faso et al., 2016; Hus et al., 2013; Sasson, et al., 2013c; Stewart et al., 2009; Takei et al., 2014), we considered that many of their items could serve to assess the BAP traits according to its updated definition.

#### Expert Judgment

Following Waltz and Bausell (1983), we took the proportion of items that received a rating of 3 or 4 by the experts as the content validity index (CVI; Hambleton et al., 1978). First, we collapsed four ordinal rating-scales into two dichotomous categories and assigned them labels (0 = content invalid, 1 = content valid). An expert agreement of 80% (indicating valid content) was taken as an index of item inclusion. Final decisions on items (i.e., conserving or deleting) were based on both data analysis and comments from the experts.

Equally, we examined (i) whether the construct of BAP was well represented by the domains and subdomains included in BAP operationalization and (ii) whether the items included in the questionnaire were sufficient to represent the construct of BAP. Furthermore, we asked the experts to suggest BAP characteristic behaviours or items that they considered relevant for improving the representation of the construct or any of its domains and subdomains.

Finally, once we had selected the items in terms of relevance to each subdomain, we verified whether the remaining items covered the entire theoretical content within the structure of BAP.

Specifically, (i) the relevance of the content of each item of the three questionnaires was studied according to the subdomain included in the BAP operationalization that showed the greatest content-based relationship, and (ii) the representativeness of a BAP test depended on the degree to which its items were proportionally distributed or weighted across the two core domains and seven subdomains (as conceptualized in alignment with the ASD definition in the APA, 2013) and whether items may cover the entire BAP construct. Considering the DSM-5 specifications of ASD deficits (APA, 2013) and the absence of any work which has highlighted that some autism deficits are more pivotal than others, we considered that the seven subdomains should be adequately represented in the final pool of items. The reason why we performed two subsequent expert judgments was due to the huge number of items. That way, a first group allocated the items and screened them to select only those with adequate psychometric properties while the second broader group confirmed that item assignment was correct and that the BAP construct was correctly represented by the items.

## Results

### Parallel Analysis

**Fig. 1:**
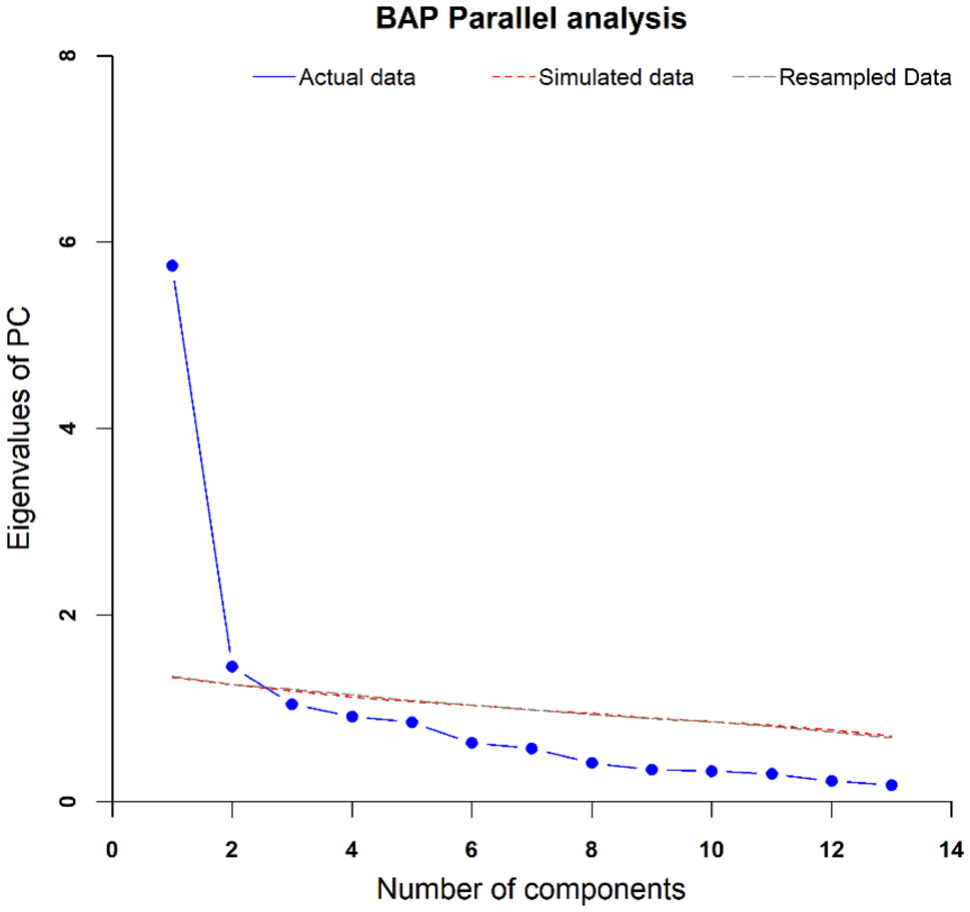
Results of Parallel Analysis Principal Components. Actual data = original data; Simulated data = random data with the same N variables and sample size; Resampled Data = repeated sample from the original data

The results of the Parallel analysis of principal components (see Fig. 1) lead us to propose that a solution of two-components underlies the covariation of the 13 BAP subscales. Furthermore, most of the variance is explained by one of these two components. The first component explained 44.23% of the total variance while the second component explained 11.15%, together they explained 55.39% of the total variance.

### Item Selection and Assignment According to Their Content and Itemmetric Properties

#### Assignment of Items According to Their Content

**Fig. 2:**
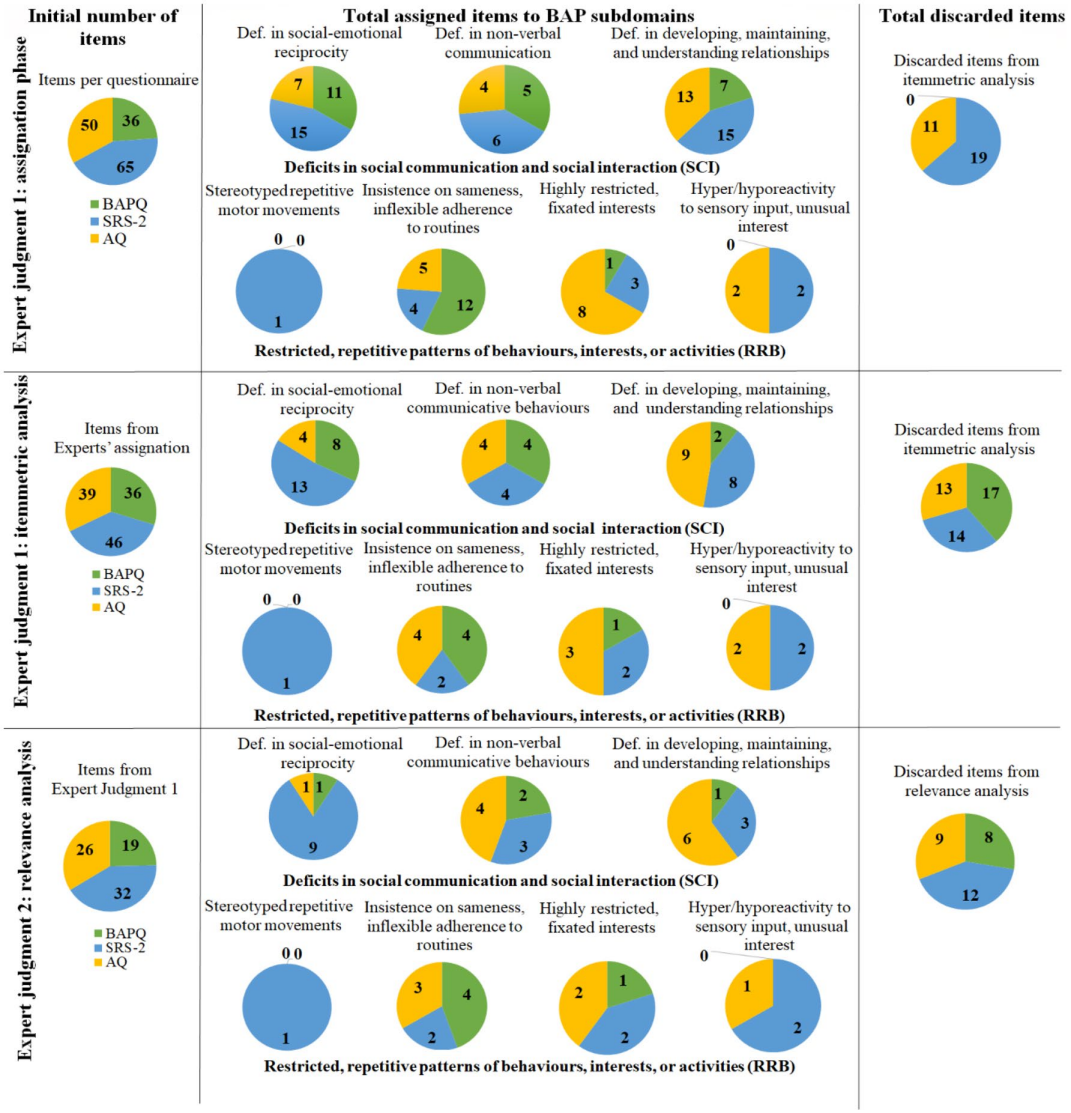
Results from both Expert Judgments

Assignment of the items to each of the seven ASD subdomains is displayed in Fig. 2. Likewise, specific items are presented in supplementary material Table 2. A total of 83 items were found to fit the SCI BAP domain while 38 fitted the RRB BAP one.

Closer inspection of the data revealed that there was also an imbalance in the distribution of the items among the subdomains. In this regard, two subdomains of RRB BAP—Stereotyped or repetitive motor movements and Hyper-or hypo-reactivity to sensory input or unusual interests, accounted for only a few items, primarily from the SRS-2. With regard to discarded items, all BAPQ items were found to fit a subdomain while 11 AQ items and 19 SRS-2 items were eliminated.

#### Itemmetric Analysis

Results from the itemmetric analysis are also shown in Fig. 2, supplementary material Tables 2, and 3. Forty-six items were eliminated by the experts (this time the discarded items belonged equally to the three questionnaires), due to a lack of clarity, comprehensibility, or concreteness, degree of self-reference, and/or the evaluation of the items. However, after reviewing the results, the experts decided to keep two items of the SRS-2 (thus 44 items were finally discarded) due to the lack of representation in Highly restricted, fixated interests, since the experts considered their contents to be key for the subdomain. Finally, RRB BAP was still the domain with the least number of items (62 SCI BAP/21 RRB BAP).

### Expert Judgment

#### The Relevance of the Items for the Subdomains

Results from Expert judgment 2 (see Fig. 2; see supplementary material Table 2) revealed a remaining pool of 48 items according to their relevance to the subdomain, 30 for SCI BAP, and 18 for RRB BAP. Stereotyped or repetitive motor movements and Hyper- or hypo-reactivity to sensory input or unusual interest subdomains in RRB BAP still accounted for few items but were maintained while the remaining subdomains of both domains now had fewer items. Twenty-nine items were discarded by the experts and these were distributed equally across the three questionnaires.

#### Representativeness of the BAP Construct

Fifteen percent of the experts rated the representation of the BAP construct as very well represented and 85% considered it well represented. Similarly, 95% of the experts considered that the items of the questionnaire represented the construct well; whilst one response was missing.

## Discussion

The purpose of this study was to connect the content of three current BAP measurement tools with an updated operationalization of the BAP harmonized with the ASD one (APA, 2013). The underlying idea was to detect possible gaps in the contents of BAP measurement instruments and to take a preliminary step towards bridging the gap between the operationalization and measurement of this phenotype.

First, we conducted a parallel analysis (Horn, 1965) to assess the factor structure of the BAP across the AQ, the BAPQ, and the SRS-2 subscales (Hurley et al., 2007; Piven, et al., 1997a, 1997b). Unlike the findings reported by Wainer et al. (2011), our results pointed to two main components instead of three. The fact that the three most applied BAP tests can cluster the variance of their 13 scales into two components provides an empirical basis for updating the BAP operationalization to a construct that uses two principal domains aligned with the current definition of ASD (APA, 2013). These results are consistent with those presented previously providing empirical evidence for the correspondence between BAP and ASD dimensions. For example, Sasson and et al., (2013c) observed that socio-communicative aspects of the BAP (measured by the Aloof and Pragmatic Language subscales of the BAPQ), unlike non-social features (measured by the BAPQ Rigid subscale) were connected to social skill and social cognition whilst also predicting poorer performance in social areas. In another study, Frazier et al. (2014) examined the SRS-2 factor structure and considered a simpler two-factor solution that showed the correspondence between SRS-2 and current ASD dimensions.

Second, we explored whether the available self-reported tests were adequate for evaluating BAP according to its updated definition. The study of the phenotype requires an adequate measurement tool that cannot disregard the actual structure and specifications of the construct. Thus, both, theoretically and empirically-based claims regarding the two-dimensional structure of the BAP (e.g., Morrison et al., 2018; Sasson, et al., 2013c) formed the basis of the main goal of this study: to reallocate its items (i.e. relevant BAP behaviours or preferences) according to the two main subdomains of ASD in the DSM-V (APA, 2013).

With respect to this allocation phase, two main observations are worth noting. All the BAPQ items were distributed across different subdomains of the proposed BAP operationalization but they did not sufficiently cover all the subdomains. That is, whilst BAPQ items might accurately evaluate subclinical autistic-like traits according to the DSM-5, the inferences based only on its items would be limited and not representative of the actual BAP construct. The SRS-2, however, with its higher number of items and contents, taps into all BAP subdomains and gathers more types of BAP-related behaviours and preferences than the BAPQ and the AQ. However, at the same time, some SRS-2 items were considered to not fit in any subdomain. Consequently, applying only the SRS-2 when measuring autism traits could cover all autistic-like features, together with some other non-autistic related deficits, which could blur the interpretation of its scores. On balance, we conclude that, when used alone, all of the proposed tests are insufficient for assessing the updated BAP operationalization. Consequently, the next step was to test whether a conjoint use of the items of the three tests would more adequately represent the BAP construct.

Third, before studying the relevance and representativeness of all these items for measuring BAP (Expert judgment 2), their wordings were scrutinized to ensure that any problem regarding their itemmetric properties would not affect the work of the experts. It is worth highlighting the importance of eliminating poorly drafted items that could yield biased responses, since these could affect the way respondents interpret and answer them (see Angleitner et al., 1986; Grant & Davis, 1997), along with the way the construct is finally assessed (Haynes et al., 1995). Furthermore, some populations, particularly BAP people, have problems when they are required to put themselves in the position of others. Consequently, they may struggle to answer items regarding others’ view of the person (non-self-referred items). As we hypothesized, some formal problems emerged during the itemmetric judgment, and half of the items were discarded in this analysis. The item property that appeared to be the most problematic was concreteness. The experts suggested that some items were too generic and/or refer to more than one idea (e.g., SRS-2 item 30 refers to more than one idea) whilst others were unclear (e.g., AQ Item 15 uses an adverb that interferes with the rating scale) or did not involve self-reference.

Fourth, we examined the relevance and representativeness of the items for covering the whole BAP construct (Haynes et al., 1995; Lynn, 1986; Nunnally & Bernstein, 1994; Suen & Ary, 1989). By doing this, the initial set of items was reduced by less than a third. In addition, both expert judgments indicated an unbalance in the distribution of items throughout the domains and subdomains where the number of items in SCI BAP was more than twice those in RRB BAP. This unbalance could be due to discrepancies in the original operationalizations of the targeted construct of each test. The BAPQ was developed based on a three-dimensional BAP structure (Hurley et al., 2007), while the AQ also included aspects of cognitive abnormality (Baron-Cohen et al., 2001). Similarly, the SRS originally aimed to assess autistic social impairment, particularly reciprocal social behaviours (Constantino & Todd, 2005) although the most recent version of this questionnaire, the SRS-2, includes a new subscale for the assessment of restricted and repetitive behaviours (Constantino & Gruber, 2012).

This asymmetric representation of domains could be challenging, given that many authors have claimed that both are important for diagnosing autism since the time that Kanner (1943) provided the first accurate definition of the disorder. Accordingly, and as now detailed in the DSM-5, the three SCI subdomains and at least two RRB subdomains must hinder the person’s everyday life to be regarded as a conclusive ASD diagnostic (APA, 2013). The same can be applied to BAP assessment such as the non-clinical expressions within the autism spectrum. Furthermore, the experts (Expert judgment 2) in our study considered that the BAP construct was well represented by the two domains and seven subdomains included in the definition we provided based on the autism spectrum definition (DSM-5; APA, 2013). These results support our claim for the need to evaluate both SCI and RRB domains equally when studying BAP.

The experts also pointed out that the selected items were representative of the construct. Although the content of the items could appear to be sufficiently representative of the BAP construct, the final allocation presents clear gaps in the BAP content. Thus, we should not disregard the underrepresentation of two RRB subdomains and its theoretical and psychometric implications. Since autism shares some indicators with other disorders, the worst scenario that could arising from neglecting certain key autistic behaviours (e.g., Hyper/hypo-reactivity to sensory input or unusual interest in sensory aspects) in preliminary test construction phases could lead to variations in final test scores that only reflect differences in traits that are also shared with other disorders.

In this regard, some studies have reported that the RRB domain can be divided into two clusters of indicators: (a) repetitive motor and sensory behaviours (repetitive hand movements) and (b) insistence on sameness (narrow interests, rigid routines, and rituals; Cuccaro et al., 2003; Honey et al., 2012; Richler et al., 2007). Assessing only one of the two RRB subtypes could lead to ASD variations being confounded with other disorders such as social communication disorder (characterised by persistent deficits in the social use of verbal and non-verbal communication in the absence of restricted and repetitive interests and behaviours; APA, 2013) or obsessive–compulsive disorder (the assessed person could not meet the criteria for the second subdomain). This would have a direct effect on BAP identification and future research studies, particularly those concerned with neurobiological and genomic aspects (Ruscio & Ruscio, 2002, 2004). For instance, there is evidence that the subdomains of RRB are underpinned by different neural pathways (Langen et al., 2011). Thus, a lack of relevant items with which to assess stereotyped or repetitive motor movements, use of objects, or speech, could negatively affect the study of the different neural pathways of autism, either in people diagnosed with autism or BAP family members.

In conclusion, researchers have begun to highlight the need to update the definition of BAP so that it is aligned with the current definition of ASD (Morrison et al., 2018; Sasson, et al., 2013c). This study represents a first step towards achieving this goal by providing empirical evidence in support of the need for a new test for evaluating the BAP that runs parallel to the ASD structure, containing its most relevant content but also including additional indicators that measure milder forms of ASD.

## Supplementary Information

Below is the link to the electronic supplementary material.

Supplementary file1 (RTF 2150 kb)

## Footnotes

1 In the present study, we used the same sample as that in Godoy-Giménez et al. (2018), where the Spanish version of the BAPQ was adapted and validity evidence was provided based on BAPQ relationships with measures of other variables: Pragmatic Awareness Questionnaire (PAQ; Rodríguez‑Muñoz, 2012) and The NEO Five‑Factor Inventory (NEO‑FFI, Spanish Version; Costa & McCrae, 1999).

2 Deficits in social-emotional reciprocity; Deficits in non-verbal communicative behaviours used for social interaction; Deficits in developing, maintaining, and understanding relationships; Stereotyped or repetitive motor movements, use of objects, or speech; Insistence on sameness, inflexible adherence to routines, or ritualized patterns of verbal or nonverbal behaviour; Highly restricted, fixated interests; and Hyper- or hypo-reactivity to sensory input or unusual interest in sensory aspects of the environment (APA, 2013).
