# Supplementary material for: Is it Possible to Assess the Two-Domain Definition of the Broad Autism Phenotype Using the Available Measurement Tools?
Source: J Autism Dev Disord. 2021 Jun 29;52(7):2884–95. doi: 10.1007/s10803-021-05158-7 (PMC9213296; doi:10.1007/s10803-021-05158-7)
Supplement: Supplementary file 1 — Supplementary file1 (RTF 2150 kb) [file 10803_2021_5158_MOESM1_ESM.rtf]

Supplementary Material for the manuscript: “Is it possible to assess the two-domain definition of the Broad Autism Phenotype using the available measurement tools?” submitted by Godoy-Giménez, M., González-Rodríguez, A., Cañadas, F1., Estévez, A. F., and Sayans-Jiménez, P. for consideration in Journal of Autism and Developmental Disorders

Supplementary Material Table 1
Table 1.
Proposed BAP operationalization	
Broad Autism Phenotype Definition (BAP)	
A.	Social interaction and social communication impairments (SCI BAP) : 
Persistent deficits in social communication and social interaction across multiple contexts.	
A1. Deficits in social-emotional reciprocity	From abnormal social approach and failure of normal back-and-forth conversation, reduced sharing of interests, emotions, or affect to failure to initiate or respond to social interactions.	
A2. Deficits in non-verbal communicative behaviours used for social interaction	From poorly integrated verbal and nonverbal communication to abnormalities in eye contact and body language, deficits in understanding and use of gestures to a total lack of facial expressions and nonverbal communication.	
A3. Deficits in developing, maintaining, and understanding relationships	From difficulties adjusting behaviours to suit various social contexts to difficulties in sharing imaginative play or to difficulties in making friends or to the absence of interest in peers.	
B.	Restricted/Repetitive Behaviours, Interests, or Activities (RRB BAP): Restricted and/or repetitive patterns of behaviours, interests, or activities.	
B1. Stereotyped or repetitive motor movements, use of objects, or speech	Simple motor stereotypes, lining up toys or flipping objects, Echolalia, Idiosyncratic phrases.	
B2. Insistence on sameness, inflexible adherence to routines, or ritualized patterns of verbal or nonverbal behaviour	Extreme distress at small changes, difficulties with transitions, rigid thinking patterns, greeting rituals, need to take the same route or eat the same food every day.	
B3. Highly restricted, fixated interests 	Hypersensitivity to criticism, excessively circumscribed or perseverative interests, strong attachment to or preoccupation with unusual objects.	
B4. Hyper- or hyporeactivity to sensory input or unusual interest in sensory aspects of the environment	Apparent indifference to pain/temperature, adverse response to specific sounds or textures, excessive smelling or touching of objects, visual fascination with lights or movement.	


Supplementary Material Table 2
Table 2.
Items selected in each phase	
A.	Social interaction and social communication impairments (SCI BAP): Persistent deficits in social communication and social interaction across multiple contexts.	
Subdomain	Test	Experts' assignation of the items	Itemmetric Analysis	Expert judgment 2	
A1. Deficits in social-emotional reciprocity	BAPQ	2, 4, 7, 11, 12, 14, 25, 27, 28, 32, 36	2, 4, 7, 11, 14, 27, 32, 36	36	
	SRS-2	3, 5, 7, 10, 12, 13, 19, 26, 34, 35, 38, 43, 48, 51, 60	3, 5, 7, 10, 12, 13, 26, 34, 35, 38, 43, 51, 60	5, 7, 10, 12, 13, 26, 35, 38, 60	
	AQ	10, 17, 18, 26, 33, 35, 38	10, 17, 33, 38	10	
A2. Deficits in non-verbal communicative behaviours used for social interaction	BAPQ	10, 20, 21, 29, 34	10, 21, 29, 34	10, 21, 34	
	SRS-2	2, 15, 16, 46, 52, 53	2, 46, 52, 53, 27,	2, 53	
	AQ	27, 31, 36, 45	31, 36, 45	27, 31, 36, 45	
A3. Deficits in developing, maintaining, and understanding relationships	BAPQ	1, 5, 9, 16, 18, 23, 31	1, 18	1	
	SRS-2	1, 6, 18, 21, 22, 23, 27, 33, 36, 37, 47, 55, 56, 57, 64	21, 22, 27, 36, 37, 47, 56, 64,	37, 47, 64,	
	AQ	1, 7, 8, 11, 15, 20, 22, 40, 42, 44, 47, 48, 50	1, 8 ,20, 40, 42, 44, 47, 48, 50	1, 20, 40, 44, 47, 50	
B.	Restricted/Repetitive Behaviours, Interests, or Activities (RRB BAP): Restricted and/or repetitive patterns of behaviours, interests, or activities.	
Subdomain	Test	Experts' assignation of the items	Itemmetric Analysis	Expert judgment 2	
B1. Stereotyped or repetitive motor movements, use of objects, or speech	BAPQ	0	0	0	
	SRS-2	50	50	50	
	AQ	0	0	0	
B2. Insistence on sameness, inflexible adherence to routines, or ritualized patterns of verbal or nonverbal behaviour	BAPQ	3, 6, 8, 13, 15, 19, 22, 24, 26, 30, 33, 35	15, 22, 24, 33	15, 22, 24, 33	
	SRS-2	4, 24, 61, 63	24, 61	24, 61	
	AQ	2, 25, 34, 43, 46	2, 25, 43, 46	2, 25, 46	
B3. Highly restricted, fixated interests 	BAPQ	17	17	17	
	SRS-2	28, 31, 39	28, 31	28, 31	
	AQ	4, 6, 9, 16, 19, 23, 39, 41	19, 39, 41	39, 41	
B4. Hyper- or hyporeactivity to sensory input or unusual interest in sensory aspects of the environment	BAPQ	0	0	0	
	SRS-2	20, 42	20, 42	20, 42	
	AQ	5, 12	5, 12	12	

Supplementary Material Table 3
Table 3.
Items discarded due to itemmetric problems in each property	
Itemmetric properties	
Questionnaire	Clarity	Comprehensibility	Concreteness	Degree of self-reference	Evaluation of the items	
BAPQ	13, 20, 25, 35	26	3, 5, 6, 8, 9, 12, 16, 19, 23, 28, 30, 31, 35	12, 26 
	5, 23	
SRS-2	4, 11, 12, 13, 18, 30, 35, 40	45	1, 4, 6, 14, 30	0	1, 4, 12, 15, 24, 36, 42, 45 	
AQ	3, 6, 13, 15, 21, 25		5, 8, 10, 11, 12, 19, 23, 25, 28 	15	6, 9, 12, 18 	


Supplementary Material Document 1  

[This page is intentionally left blank]


CUESTIONARIO DE EXPERTOS/AS PARA LA
CONSTRUCCIÓN DE UN TEST INTEGRADO DEL
FENOTIPO AMPLIADO AUTISTA
Guía y conceptos básicos.


Es recomendable tener esta guía al alcance a medida que se contesta el cuestionario, en ella vienen recogidas las instrucciones necesarias e información complementaria para dicha tarea.


Carta de presentación

Distinguido/a profesional:
Nos dirigimos a usted dada su amplia experiencia con personas diagnosticadas con Trastornos del Espectro Autista (TEA) y con familiares de los mismos. El objeto de esta comunicación es solicitar su colaboración, en calidad de experto/a, para la construcción del primer instrumento de medida de las expresiones del Fenotipo Ampliado del Autismo (FAA) en la población española.  Para ello, el primer paso consiste en seleccionar de entre las herramientas más relevantes a nivel internacional, cuáles serían los ítems más adecuados para captar las dimensiones y subdimensiones del TEA definidas en el DSM-V pero aplicadas a población subclínica española. 
Antes de continuar, permítanos definirle el concepto de FAA; el FAA hace referencia a un conjunto de características subclínicas cualitativamente similares a aquellas presentadas por las personas diagnosticadas con Trastorno del Espectro Autista (TEA), pero más sutiles en su expresión. Inicialmente, el FAA fue observado en padres de niños ya diagnosticados con TEA y en familiares de primer orden (cercanos). Sin embargo, recientes estudios apoyan su existencia también en población general. En la actualidad, son tres los instrumentos utilizados en la literatura para medir FAA: el Autism Quotient (AQ; Baron-Cohen et al., 2001), el Social Responsiveness Scale (SRS; Constantino, 2007), y el Broad Autism Phenotype questionnaire (BAPQ; Hurley et al., 2007). Sin embargo, estas herramientas de medida o bien no se encuentran disponibles para su aplicación en la población española o bien no presentan adecuadas propiedades psicométricas. 
 Consecuentemente, en nuestro estudio nos proponemos crear una nueva herramienta para la medición del FAA en sintonía con la nueva conceptualización del DSM-V (APA, 2013) y adaptada a la población española, a partir de la selección de los ítems más adecuados de los cuestionarios ya mencionados. El Objetivo General para el que se empleará el test será el de diferenciar entre distintitos grados de severidad de FAA en cada una de las subdimensiones del TEA, tanto en familiares de niños diagnosticados con TEA como en población general.
Su ayuda es un pilar fundamental en esta fase del estudio. Solo una adecuada muestra de expertos/as en todas las áreas que tratan con personas diagnosticadas con TEA, o con características salientes del FAA, permitirá seleccionar de forma neutral y precisa cuáles son los ítems más adecuados para llevar a cabo una medición eficiente y fiable que permita la detección de los rasgos más sutiles del FAA.
Dada la importancia de su colaboración, además de mostrarle nuestro más sincero agradecimiento, nos gustaría ponernos a su disposición para cualquier tipo de consulta, colaboración o ayuda que podamos brindarle desde nuestro equipo de investigación. Adicionalmente, nos gustaría indicarle que, si así lo desea, le mantendremos informado sobre el desarrollo de la herramienta de medida y le facilitaremos dicha herramienta y su baremo una vez hayamos finalizado el proceso de construcción. Asimismo, como no podría ser menos, en caso de que finalmente decida colaborar con nuestro equipo, su nombre será incluido en los agradecimientos del artículo de investigación que describa el proceso de construcción de la herramienta de medida.

Atentamente,


M. Ángeles Estévez, investigadora principal del proyecto, perteneciente al grupo de investigación HUM891 (Investigación en Neurociencia Cognitiva) y profesora titular de la Universidad de Almería.


Instrucciones para llevar a cabo el juicio de expertos
Para llevar a cabo el siguiente procedimiento es necesario que usted tenga claros los siguientes aspectos:
1.	La población a la que está dirigida el test es tanto población general como familiares de personas diagnosticadas con TEA.
2.	El objetivo último del test es poder diferenciar, a las personas que contesten al test, en función de la severidad con la que expresan las características principales de cada subdimensión (sin olvidar que estas expresiones se expresarán sutilmente o a nivel subclínico).
3.	La estructura del test que se pretende desarrollar coincide con la definición del constructo del TEA en el DSM-V (APA, 2013): dos dimensiones principales con tres y cuatro subdimensiones respectivamente.

TABLA DE CONTENIDO	
	Dimensión		Subdimensión	
Problemas en la comunicación e interacción social	1)	Deficiencias en la reciprocidad socioemocional (es decir, problemas en la interacción y comunicación socioemocional).	
	2)	Deficiencias en las conductas comunicativas no verbales utilizadas en la interacción social.	
	3)	Deficiencias en el desarrollo, mantenimiento y comprensión de las relaciones (es decir, saber cómo comportarse en diferentes situaciones sociales, dificultades para hacer amigos y ausencia de interés por otras personas).	
Patrones de intereses obsesivos y conductas repetitivas	1)	Movimientos, utilización de objetos o habla estereotipados o repetitivos.	
	2)	Insistencia en la monotonía, excesiva inflexibilidad de rutinas o patrones  ritualizados de comportamiento verbal o no  verbal.	
	3)	Intereses muy restringidos y fijos que son anormales en cuanto a su intensidad o foco de interés.	
	4)	Híper o hiporreactividad a los estímulos sensoriales o interés inhabitual por aspectos sensoriales del entorno.	


4.	La interpretación de las puntuaciones del test se llevará a cabo usando modelos de teoría de respuesta al ítem. No estamos buscando ítems que sean paralelos en cada subdimensión (que midan exactamente lo mismo). Pretendemos incluir ítems que expresen distinto grado de severidad en cada subdimensión. 
5.	Cada vez que emita un juicio o conteste a una pregunta referida al test tiene que tener en mente la población a la que está dirigido (tanto población general como familiares de personas diagnosticadas con TEA) y el objetivo último del test (diferenciar, a nivel subclínico, a las personas que contesten al test en función de la severidad con la que expresan cada subdimensión).
6.	Tendrá que realizar juicios sobre el test, sus instrucciones, los ítems y sus opciones de respuesta basados en los siguientes conceptos:
a.	Relevancia: importancia/relación con la subdimensión destacada. Esta característica indica en qué medida el ítem refleja comportamientos o indicadores destacados de cada subdimensión.
b.	Adecuación a los objetivos: grado en el que cumple las necesidades del estudio. Cada ítem pretende diferenciar a las personas que tengan distintos niveles de FAA expresados específicamente en cada subdimensión de la forma más eficiente posible (es importante recordar que la población destino será población general o familiares de niños/as diagnosticados con TEA, es decir, personas cuya mayor severidad en la expresión del TEA será a nivel subclínico). En los ítems poco adecuados todas las personas responderán casi de la misma manera (generalmente las categorías centrales), independientemente de sus niveles de FAA.
c.	Respuesta diferencial en función de distintos grupos: grado en el que el contenido del ítem puede generar respuestas diferentes SOLO debidas al grupo de pertenencia (o bien población general, o bien padres/familiares de niños/as diagnosticados con TEA) y no debidas a los distintos niveles de FAA de las personas que contestan.


Supplementary Material Document 2

[This page is intentionally left blank]


Questionario de expertos/as para la construcción de un test integrado del Fenotipo Ampliado Autista


Estudio sobre la construcción de una nueva herramienta de medida que evalúe FAA en población española.
	


[This page is intentionally left blank]


DIMENSIÓN 1: PROBLEMAS EN LA COMUNICACIÓN E INTERACCIÓN SOCIAL.	
SUBDIMENSIÓN 1: DEFICIENCIAS EN LA REPCIPROCIDAD SOCIOEMOCIONAL 	
Opciones de respuesta: 1 (ninguna), 2 (leve), 3 (moderada) y 4 (totalmente).	
Ítem	Relevancia para la sub-dimensión	Adecuación a los objetivos	Respuesta diferencial en función de distintos grupos	
1a	Al conversar con otras personas me siento fuera de lugar.	1	2	3	4	1	2	3	4	1	2	3	4	
1b	Disfruto estando de cháchara (hablando de trivialidades).	1	2	3	4	1	2	3	4	1	2	3	4	
1c	Tengo dificultades para responder preguntas de manera directa y acabo yéndome por las ramas.	1	2	3	4	1	2	3	4	1	2	3	4	
1d	Mientras mantenemos una conversación congenio con la otra persona.	1	2	3	4	1	2	3	4	1	2	3	4	
1e	Disfruto de las conversaciones triviales (conversaciones informales con otras personas).	1	2	3	4	1	2	3	4	1	2	3	4	
1f	Dar conversación me aburre.	1	2	3	4	1	2	3	4	1	2	3	4	
1g	Disfruto charlando con la gente.	1	2	3	4	1	2	3	4	1	2	3	4	
1h	Normalmente soy consciente de cómo se sienten los demás.	1	2	3	4	1	2	3	4	1	2	3	4	
1i	Evito a la gente que intenta acercarse emocionalmente a mí.	1	2	3	4	1	2	3	4	1	2	3	4	
1j	Me cuesta seguir el curso de las conversaciones.	1	2	3	4	1	2	3	4	1	2	3	4	
1k	La gente me pide que repita lo que he dicho porque no han entendido lo que he querido decir.	1	2	3	4	1	2	3	4	1	2	3	4	
1l	Me siento seguro/a de mí mismo/a cuando interactúo con otras personas.	1	2	3	4	1	2	3	4	1	2	3	4	
1m	Cuando hablo con la gente tengo tendencia a irme por las ramas.	1	2	3	4	1	2	3	4	1	2	3	4	


DIMENSIÓN 1: PROBLEMAS EN LA COMUNICACIÓN E INTERACCIÓN SOCIAL.	
SUBDIMENSIÓN 1: DEFICIENCIAS EN LA REPCIPROCIDAD SOCIOEMOCIONAL 	
Opciones de respuesta: 1 (ninguna), 2 (leve), 3 (moderada) y 4 (totalmente).	
Ítem	Relevancia para la sub-dimensión	Adecuación a los objetivos	Respuesta diferencial en función de distintos grupos	
1n	Respondo de manera apropiada a los cambios de humor de los demás (e.g., cuando un amigo pasa de estar contento a estar triste).	1	2	3	4	1	2	3	4	1	2	3	4	
1o	En situaciones sociales, tengo facilidad para seguir el hilo de diferentes conversaciones.	1	2	3	4	1	2	3	4	1	2	3	4	
1p	En una conversación telefónica, no estoy seguro/a de cuándo me toca hablar.	1	2	3	4	1	2	3	4	1	2	3	4	
1q	Tengo problemas para mantener el curso de una conversación.	1	2	3	4	1	2	3	4	1	2	3	4	
1r	Ofrezco consuelo a los demás cuando se sienten tristes.	1	2	3	4	1	2	3	4	1	2	3	4	
1s	Los demás piensan que soy emocionalmente distante y que no muestro mis sentimientos.	1	2	3	4	1	2	3	4	1	2	3	4	
1t	Se me da bien la cháchara (hablar de trivialidades).	1	2	3	4	1	2	3	4	1	2	3	4	
1u	Soy capaz de expresar mis sentimientos a otras personas.	1	2	3	4	1	2	3	4	1	2	3	4	
1v	Soy torpe con los turnos en las interacciones con los demás (e.g., me cuesta trabajo mantener. El “toma y daca de una conversación).	1	2	3	4	1	2	3	4	1	2	3	4	
1w	Me tomo las cosas al pie de la letra, y por eso malinterpreto el significado de algunas partes de la conversación.	1	2	3	4	1	2	3	4	1	2	3	4	
1x	Me resulta difícil expresarme con fluidez.	1	2	3	4	1	2	3	4	1	2	3	4	
1y	No me doy cuenta cuándo los demás están tratando de aprovecharse de mí.	1	2	3	4	1	2	3	4	1	2	3	4	


-	¿Considera que alguno de los anteriores ítems podría ser más adecuado para medir cualquier otra subdimensión distinta de esta? Consulte la Tabla de contenido del apartado 3 de las Instrucciones para llevar a cabo el juicio de expertos y rodee la respuesta correcta.
·	No.
·	Sí. 

-	En caso de que así sea ¿qué subdimensión/es sería/n?
         Ítem
       (1a, 1b, 1c…)		Subdimensión	
			
			
			
			
			
			
			


DIMENSIÓN 1: PROBLEMAS EN LA COMUNICACIÓN E INTERACCIÓN SOCIAL.	
SUBDIMENSIÓN 2: DEFICIENCIAS EN LAS CONDUCTAS COMUNICATIVAS NO VERBALES UTILIZADAS EN LA INTERACCIÓN SOCIAL	
Opciones de respuesta: 1 (ninguna), 2 (leve), 3 (moderada) y 4 (totalmente).	
Ítem	Relevancia para la sub-dimensión	Adecuación a los objetivos	Respuesta diferencial en función de distintos grupos	
2a	Durante una conversación con otras personas suelo dejar largas pausas.	1	2	3	4	1	2	3	4	1	2	3	4	
2b	Me resulta fácil “leer entre líneas” cuando alguien me habla.	1	2	3	4	1	2	3	4	1	2	3	4	
2c	Soy capaz de darme cuenta si una persona que me está escuchando se aburre.	1	2	3	4	1	2	3	4	1	2	3	4	
2d	Levanto la voz demasiado sin darme cuenta.	1	2	3	4	1	2	3	4	1	2	3	4	
2e	Me resulta fácil adivinar lo que alguien está pensando o sintiendo con sólo mirarle a la cara.	1	2	3	4	1	2	3	4	1	2	3	4	
2f	Cuando converso con alguien mi voz tiene una entonación plana o monótona.	1	2	3	4	1	2	3	4	1	2	3	4	
2g	Los demás piensan que mis expresiones faciales son demasiado serias.	1	2	3	4	1	2	3	4	1	2	3	4	
2h	Tiendo a hablar con una voz monótona (en otras palabras, con menos cambios en la voz que la mayoría de la gente).	1	2	3	4	1	2	3	4	1	2	3	4	
2i	Mis expresiones faciales proporcionan información errónea a los demás acerca de cómo me siento.	1	2	3	4	1	2	3	4	1	2	3	4	
2j	Sé cuando alguien no está interesado en lo que estoy diciendo.	1	2	3	4	1	2	3	4	1	2	3	4	
2k	Cuando estoy conversando me doy cuenta de cuándo es el momento de cambiar de tema.	1	2	3	4	1	2	3	4	1	2	3	4	
2l	Me resulta difícil adivinar las intenciones de los demás.	1	2	3	4	1	2	3	4	1	2	3	4	


-	¿Considera que alguno de los anteriores ítems podría ser más adecuado para medir cualquier otra subdimensión distinta de esta? Consulte la Tabla de contenido del apartado 3 de las Instrucciones para llevar a cabo el juicio de expertos y rodee la respuesta correcta.
·	No.
·	Sí. 

-	En caso de que así sea ¿qué subdimensión/es sería/n?
         Ítem
       (2a, 2b, 2c…)		Subdimensión	
			
			
			
			
			
			
			


DIMENSIÓN 1: PROBLEMAS EN LA COMUNICACIÓN E INTERACCIÓN SOCIAL.	
SUBDIMENSIÓN 3: DEFICIENCIAS EN EL DESARROLLO, MANTENIMIENTO Y COMPRENSIÓN DE LAS RELACIONES.	
Opciones de respuesta: 1 (ninguna), 2 (leve), 3 (moderada) y 4 (totalmente).	
Ítem	Relevancia para la sub-dimensión	Adecuación a los objetivos	Respuesta diferencial en función de distintos grupos	
3a	Cuando leo una historia, tengo facilidad para imaginarme cómo podría ser el aspecto de los personajes.	1	2	3	4	1	2	3	4	1	2	3	4	
3b	Soy capaz de imitar las acciones o expresiones de otros cuando es socialmente apropiado hacer.	1	2	3	4	1	2	3	4	1	2	3	4	
3c	Cuando leo una historia, encuentro difícil adivinar las intenciones de los personajes.	1	2	3	4	1	2	3	4	1	2	3	4	
3d	Interactúo de manera apropiada con otros adultos.	1	2	3	4	1	2	3	4	1	2	3	4	
3e	Soy una persona diplomática.	1	2	3	4	1	2	3	4	1	2	3	4	
3f	A veces cometo el error de caminar entre dos personas que están tratando de hablar entre ella.	1	2	3	4	1	2	3	4	1	2	3	4	
3g	Me resulta muy fácil jugar con niños/as a juegos simbólicos o inventados.	1	2	3	4	1	2	3	4	1	2	3	4	
3h	Tengo dificultades para relacionarme con adultos que no sean de mi familia.	1	2	3	4	1	2	3	4	1	2	3	4	
3i	Tengo dificultades para relacionarme con los miembros de mi familia.	1	2	3	4	1	2	3	4	1	2	3	4	
3j	Me gusta estar con otras personas.	1	2	3	4	1	2	3	4	1	2	3	4	
3k	Me cuesta imaginarme cómo sería ser otra persona.	1	2	3	4	1	2	3	4	1	2	3	4	
3l	Disfruto estando en situaciones sociales.	1	2	3	4	1	2	3	4	1	2	3	4	


DIMENSIÓN 1: PROBLEMAS EN LA COMUNICACIÓN E INTERACCIÓN SOCIAL.	
SUBDIMENSIÓN 3: DEFICIENCIAS EN EL DESARROLLO, MANTENIMIENTO Y COMPRENSIÓN DE LAS RELACIONES.	
Opciones de respuesta: 1 (ninguna), 2 (leve), 3 (moderada) y 4 (totalmente).	
Ítem	Relevancia para la sub-dimensión	Adecuación a los objetivos	Respuesta diferencial en función de distintos grupos	
3m	Cuando era un niño/a, solía pasármelo bien jugando con otros/as niños/as a juegos simbólicos o inventados.	1	2	3	4	1	2	3	4	1	2	3	4	
3n	Evito comenzar interacciones sociales con otros adultos.	1	2	3	4	1	2	3	4	1	2	3	4	
3o	Me siento mucho más tenso/a en ambientes sociales que cuando estoy solo/a.	1	2	3	4	1	2	3	4	1	2	3	4	
3p	Prefiero hacer cosas con otras personas en vez de hacerlas solo/a.	1	2	3	4	1	2	3	4	1	2	3	4	
3q	Disfruto conociendo gente nueva.	1	2	3	4	1	2	3	4	1	2	3	4	
3r	Cuando doy conversación es sólo para ser educado/a.	1	2	3	4	1	2	3	4	1	2	3	4	
3s	Me río en momentos inapropiados.	1	2	3	4	1	2	3	4	1	2	3	4	
	


-	¿Considera que alguno de los anteriores ítems podría ser más adecuado para medir cualquier otra subdimensión distinta de esta? Consulte la Tabla de contenido del apartado 3 de las Instrucciones para llevar a cabo el juicio de expertos y rodee la respuesta correcta.
·	No.
·	Sí. 

-	En caso de que así sea ¿qué subdimensión/es sería/n?
         Ítem
       (3a, 3b, 3c…)		Subdimensión	
			
			
			
			
			
			
			


DIMENSIÓN 2: PATRONES RESTRICTIVOS Y REPETITIVOS DE COMPORTAMIENTO, INTERESES O ACTIVIDADES.	
SUBDIMENSIÓN 1: PATRONES RESTRICTIVOS Y REPETITIVOS DE COMPORTAMIENTO, INTERESES O ACTIVIDADES.	
Opciones de respuesta: 1 (ninguna), 2 (leve), 3 (moderada) y 4 (totalmente).	
Ítem	Relevancia para la sub-dimensión	Adecuación a los objetivos	Respuesta diferencial en función de distintos grupos	
4a Tengo comportamientos repetitivos que los demás consideran extraños.	1	2	3	4	1	2	3	4	1	2	3	4	

-	¿Considera que este ítem (4a) podría ser más adecuado para medir cualquier otra subdimensión distinta de esta? Consulte la Tabla de contenido del apartado 3 de las Instrucciones para llevar a cabo el juicio de expertos y rodee la respuesta correcta.
·	No.
·	Sí. 

-	En caso de que así sea ¿qué subdimensión sería?
Subdimensión	
	
	
	
	


DIMENSIÓN 2: PATRONES RESTRICTIVOS Y REPETITIVOS DE COMPORTAMIENTO, INTERESES O ACTIVIDADES.	
SUBDIMENSIÓN 2: INSISTENCIA EN LA MONOTONÍA, EXCESIVA INFLEXIBILIDAD DE RUTINAS O PATRONES  RITUALIZADOS DE COMPORTAMIENTO VERBAL O NO VERBAL.	
Opciones de respuesta: 1 (ninguna), 2 (leve), 3 (moderada) y 4 (totalmente).	
Ítem	Relevancia para la sub-dimensión	Adecuación a los objetivos	Respuesta diferencial en función de distintos grupos	
5a	Me gusta planificar minuciosamente cualquier actividad en la que participo.	1	2	3	4	1	2	3	4	1	2	3	4	
5b	Me gusta seguir una rutina minuciosa cuando trabajo.	1	2	3	4	1	2	3	4	1	2	3	4	
5c	Prefiero hacer las cosas siempre de la misma manera.	1	2	3	4	1	2	3	4	1	2	3	4	
5d	Las situaciones novedosas me producen ansiedad.	1	2	3	4	1	2	3	4	1	2	3	4	
5e	Soy flexible sobre cómo deberían hacerse las cosas.	1	2	3	4	1	2	3	4	1	2	3	4	
5f	Tiendo a ser inflexible.  	1	2	3	4	1	2	3	4	1	2	3	4	
5g	No me molesta que mi rutina diaria se vea alterada.	1	2	3	4	1	2	3	4	1	2	3	4	
5h	Soy muy rígido/a en mis costumbres.	1	2	3	4	1	2	3	4	1	2	3	4	
5i	Tengo más dificultad que los demás para aceptar los cambios que se producen en mi rutina.	1	2	3	4	1	2	3	4	1	2	3	4	
5j	Tengo dificultades para enfrentarme a cambios en mi rutina.	1	2	3	4	1	2	3	4	1	2	3	4	


-	¿Considera que alguno de los anteriores ítems podría ser más adecuado para medir cualquier otra subdimensión distinta de esta? Consulte la Tabla de contenido del apartado 3 de las Instrucciones para llevar a cabo el juicio de expertos y rodee la respuesta correcta.
·	No.
·	Sí. 

-	En caso de que así sea ¿qué subdimensión/es sería/n?
         Ítem
       (5a, 5b, 5c…)		Subdimensión	
			
			
			
			
			
			
			


DIMENSIÓN 3: PATRONES RESTRICTIVOS Y REPETITIVOS DE COMPORTAMIENTO, INTERESES O ACTIVIDADES.	
SUBDIMENSIÓN 3: INTERESES MUY RESTRINGIDOS Y FIJOS QUE SON ANORMALES EN CUANTO A SU INTENSIDAD O FOCO DE INTERÉS.	
Opciones de respuesta: 1 (ninguna), 2 (leve), 3 (moderada) y 4 (totalmente).	
Ítem	Relevancia para la sub-dimensión	Adecuación a los objetivos	Respuesta diferencial en función de distintos grupos	
6a	Me han dicho que hablo demasiado sobre ciertos temas.	1	2	3	4	1	2	3	4	1	2	3	4	
6b	Me gusta recopilar información sobre diferentes categorías de objetos (e.g., tipos de coches, de pájaros, de trenes, de plantas, etc.)	1	2	3	4	1	2	3	4	1	2	3	4	
6c	Pienso o hablo sobre el mismo tema una y otra vez.	1	2	3	4	1	2	3	4	1	2	3	4	
6d	Me fascinan los números.	1	2	3	4	1	2	3	4	1	2	3	4	
6e	A menudo la gente me dice que vuelvo una y otra vez sobre el mismo tema.	1	2	3	4	1	2	3	4	1	2	3	4	
6f	Una vez que empiezo a pensar en algo, no puedo sacármelo de la cabeza.	1	2	3	4	1	2	3	4	1	2	3	4	


-	¿Considera que alguno de los anteriores ítems podría ser más adecuado para medir cualquier otra subdimensión distinta de esta? Consulte la Tabla de contenido del apartado 3 de las Instrucciones para llevar a cabo el juicio de expertos y rodee la respuesta correcta.
·	No.
·	Sí. 

-	En caso de que así sea ¿qué subdimensión/es sería/n?
         Ítem
       (6a, 6b, 6c…)		Subdimensión	
			
			
			
			
			
			
			


DIMENSIÓN 3: PATRONES RESTRICTIVOS Y REPETITIVOS DE COMPORTAMIENTO, INTERESES O ACTIVIDADES.	
SUBDIMENSIÓN 4: HIPER O HIPORREACTIVIDAD A LOS ESTÍMULOS SENSORIALES O INTERESES INHABITUALES POR ASPECTOS SENSORIALES DEL ENTORNO.	
Opciones de respuesta: 1 (ninguna), 2 (leve), 3 (moderada) y 4 (totalmente).	
Ítem	Relevancia para la sub-dimensión	Adecuación a los objetivos	Respuesta diferencial en función de distintos grupos	
7a	A menudo percibo sonidos débiles que los demás no escuchan.	1	2	3	4	1	2	3	4	1	2	3	4	
7b	Tengo intereses sensoriales que a los demás les parecen inusuales (e.g., oler o mirar las coas de una manera especial).	1	2	3	4	1	2	3	4	1	2	3	4	
7c	Suelo darme cuenta de detalles que otras personas pasan por alto.	1	2	3	4	1	2	3	4	1	2	3	4	
7d	Soy excesivamente sensible a ciertos sonidos, texturas u olores.	1	2	3	4	1	2	3	4	1	2	3	4	

	¿Considera que alguno de los anteriores ítems podría ser más adecuado para medir cualquier otra subdimensión distinta de esta? Consulte la Tabla de contenido del apartado 3 de las Instrucciones para llevar a cabo el juicio de expertos y rodee la respuesta correcta.
·	No.
·	Sí. 
-	En caso de que así sea ¿qué subdimensión/es sería/n?
         Ítem
       (7a, 7b, 7c…)		Subdimensión	
			
			


BLOQUE 2: Propiedades del test
Para finalizar nos gustaría que evaluara algunas de las propiedades del test en su conjunto (incluyendo todos los ítems y subdimensiones anteriormente mencionadas).
8.-	¿En qué grado considera que el constructo de FAA está adecuadamente representado mediante las SUBDIMENSIONES empleadas? Rodee la opción que considere más adecuada.
1
Muy mal representado	2
Mal 
representado	3
Bien 
representado	4
Muy bien representado	

9.-	En caso de que considere que el constructo de FAA no se encuentre adecuadamente representado por las subdimensiones empleadas ¿qué dimensiones o subdimensiones considera usted que deberían incluirse?

Dimensión	Subdimensiones	
	

	
	

	
	

	
(En caso de que necesite espacio adicional, este cuestionario incluye un bloque de comentarios libres y una página en blanco al final para añadir las consideraciones que usted desee).

10.-	¿En qué medida cree que el constructo de FAA está adecuadamente representado mediante los ÍTEMS empleados? Rodee la opción que considere más adecuada.

1
Muy mal representado	2
Mal 
representado	3
Bien 
representado	4
Muy bien representado	


11.-	En caso de que considere que los ítems presentados NO representen adecuadamente el dominio de FAA, teniendo en cuenta los objetivos y la población a la que va dirigida el test, ¿qué tipo comportamientos o ítems referidos a los mismos cree usted que sería necesario añadir?

Subdimensión	Ítems/comportamientos que sería necesario añadir	
Deficiencias en la reciprocidad socioemocional.	


	
Deficiencias en las conductas comunicativas no verbales utilizadas en la interacción social.	


	
Deficiencias en el desarrollo, mantenimiento y comprensión de las relaciones.	


	
Movimientos, utilización de objetos o habla estereotipados o repetitivos.	


	
Insistencia en la monotonía, excesiva inflexibilidad de rutinas o patrones ritualizados de comportamiento verbal o no verbal.	


	
Intereses muy restringidos y fijos que son anormales en cuanto a su intensidad o foco de interés.	


	
Híper o hiporreactividad a los estímulos sensoriales o interés inhabitual por aspectos sensoriales del entorno.	


	

(En caso de que necesite espacio adicional, este cuestionario incluye un bloque de comentarios libres y una página en blanco al final ara añadir las consideraciones que usted desee).


12.-	A continuación se le presenta cuál será el formato de respuesta para cada uno de los ítems:


1
Muy poco adecuado	2
Poco
adecuado	3
Adecuado	4
Muy
adecuado	
¿En qué medida considera usted que el formato de respuesta es adecuado según los objetivos del test y la población a la que éste está destinado? Rodee la opción que considere más adecuada.


13.-	A continuación se le muestran las instrucciones que se incluyen en el cuestionario:


1
Muy poco adecuadas	2
Poco
adecuadas	3
Adecuadas	4
Muy
adecuadas	
Por favor, indique en qué medida considera usted que las instrucciones del test son adecuadas según los objetivos del mismo y la población a la que está destinado. Rodee la opción que considere más adecuada.


BLOQUE 3: Sugerencias, comentarios y agradecimiento
A continuación, nos gustaría facilitarle un espacio donde usted pueda expresar libremente su opinión general acerca del test, del procedimiento de juicio de expertos o de cualquier otra cosa que estime oportuna que el equipo de investigación deba tener en cuenta.


Finalmente, nos gustaría agradecer con toda sinceridad su inestimable ayuda y colaboración con el desarrollo de nuestra investigación encaminada a construir primer test en español para medir expresiones del Fenotipo Ampliado del Autismo. 
El equipo investigador: Fernando Cañadas Pérez, M. Ángeles Fernández Estévez, Marta Godoy Giménez, Antonio González Rodríguez, Pablo Sayans Jiménez.


Datos adicionales.
	La información recogida en todas las preguntas que se presentan a continuación se procesará de forma independiente de las respuestas ofrecidas en el cuestionario de expertos. El objetivo del siguiente bloque es garantizar que el panel de expertos cubre todos los ámbitos profesionales relacionados con TEA y/o FAA. Además, le solicitamos información para que, de así desearlo, podamos enviarle el cuestionario en su versión final e incluirle en los agradecimientos de la publicación donde figuren los resultados del juicio de expertos. 
Por favor, indique, mediante una X, en cuál de los siguientes ámbitos desarrolla su labor profesional y especifique, en la medida de lo posible, cuál es su perfil profesional (solo puede escoger un ámbito).
˜	Ámbito sanitario.
˜	Atención temprana (Psicólogo, Logopeda, Neuropsicólogo, Neuropediatra)
Perfil profesional: ______________________________
˜	Psicólogo clínico (PIR)
Perfil profesional: ______________________________
˜	Psiquiatra
Perfil profesional: ______________________________
˜	Psicólogo Sanitario (MPGS o licenciado convalidado)
Perfil profesional: ______________________________
˜	Ámbito social.
˜	Actuación de los Servicios Sociales en la promoción social de la Familia
Perfil profesional: ______________________________
˜	Asociaciones de TEA o familiares
Perfil profesional: ______________________________
˜	Ámbito educativo
˜	Equipos de orientación educativa
Perfil profesional: ______________________________
˜	Pedagogía terapéutica
Perfil profesional: ______________________________
˜	Centros específicos
Perfil profesional: ______________________________
˜	Ámbito académico
˜	Área y perfil investigador
En caso de que usted quiera aparecer en los agradecimientos o quiera recibir la versión final del cuestionario (este proceso se puede demorar entre uno y dos años) facilite a continuación la información de contacto donde quiera que le sea enviado.
__________________________________________________________________________________________________________________________________________________________________________________________________________________
Por favor, una vez cumplimentado, introduzca ese documento en el sobre adjunto y ciérrelo. Gracias.
